# Structural and magnetic properties of ternary Fe_1–_*_x_*Mn*_x_*Pt nanoalloys from first principles

**DOI:** 10.3762/bjnano.2.20

**Published:** 2011-03-16

**Authors:** Markus E Gruner, Peter Entel

**Affiliations:** 1 Faculty of Physics and Center for Nanointegration, CeNIDE, University of Duisburg-Essen, D-47048 Duisburg, Germany

**Keywords:** density functional theory, Fe–Pt, magnetic data recording, magnetostructural transition, Mn–Pt

## Abstract

**Background:** Structural and magnetic properties of binary Mn–Pt and ternary Fe_1–_*_x_*Mn*_x_*Pt nanoparticles in the size range of up to 2.5 nm (561 atoms) have been explored systematically by means of large scale first principles calculations in the framework of density functional theory. For each composition several magnetic and structural configurations have been compared.

**Results:** The concentration dependence of magnetization and structural properties of the ternary systems are in good agreement with previous bulk and thin film measurements. At an intermediate Mn-content around *x* = 0.25 a crossover between several phases with magnetic and structural properties is encountered, which may be interesting for exploitation in functional devices.

**Conclusion:** Addition of Mn effectively increases the stability of single crystalline L1_0_ particles over multiply twinned morphologies. This, however, compromises the stability of the ferromagnetic phase due to an increased number of antiferromagnetic interactions. The consequence is that only small additions of Mn can be tolerated for data recording applications.

## Introduction

Magnetic transition metal alloy nanoparticles provide a large variety of possibilities in several technological fields, such as biomedical diagnostics or therapy, catalysis or even mechanical actuation [1–9] and have become the focus of much research. Another application, widely discussed in recent years, is in the field of ultra-high density magnetic recording. Here, an exponential increase in storage density has been encountered over a long period of time keeping apace with the analogous development in semiconductor technology known as Moore’s law. A further continuation of this trend by increasing miniaturization, however, is threatened by hard to surmount physical limitations. Probably the most severe is the so-called superparamagnetic limit. This derives from the fact that the Néel relaxation law, which relates the relaxation time τ of the magnetization to the exponential of the product of anisotropy constant *K*_u_ times the grain volume *V* divided by temperature:

[1]
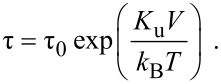


This imposes a lower boundary on the possible size of a grain made of a specific material, as this is supposed to keep its magnetization direction at ambient conditions unaffected by thermal relaxation for a sufficient amount of time, which is essentially given by τ.

Therefore, it is deemed necessary to switch to new types of recording media in future. Two concepts have been extensively discussed in this context: The first is to abandon contemporary polycrystalline media consisting of several tens or hundreds of loosely coupled grains per bit, each of them being subject to Néels relaxation law (Equation 1) and switch to a patterned medium where one bit is essentially represented by one single crystalline dot or nanoparticle [10–12]. Another promising strategy to obtain a substantial increase in integration density is to improve the materials constant *K*_u_, which together with the particle volume is part of the exponential and thus allows a very effective way of decreasing *V* [13–14]. The most promising materials in this respect are probably L1_0_ ordered FePt and CoPt [7,13,15–18]. For these materials, hypothetical lower limits for the particle diameters can be derived from Equation (1) being as small as 3 nm if the bulk values of the anisotropy constant are assumed. Both materials owe their large magnetocrystalline anisotropy to the strong hybridization of the electronic states of the 3d and 5d elements [19]. In addition, the L1_0_ order, which is defined by a layer-wise alternating stacking of the elements along the [001] direction, reduces the cubic symmetry to tetragonal and thus allows for large uniaxial contributions: The lattice sites are characterized by a tetragonally distorted face centered cubic coordination with a slightly shortened *c* axis. However, the corresponding phase with cubic symmetry is described by the CsCl structure (B2), possessing a bcc-type rather than a fcc-type coordination. Thus the effective tetragonal distortion can be deemed to be quite large.

In reality, the ad hoc extrapolation from Equation (1) transpires to be of limited applicability as it has been discovered that L1_0_ particles with a sufficient magnetocrystalline anisotropy are difficult to obtain in the corresponding size range [16,20–23]. It is certainly a straight-forward idea to seek the problem in the lower dimensionality of the particles. These naturally contain a significant percentage of surface atoms with diameters of a few nanometers. Several authors have therefore argued that at small particle sizes ordering is suppressed due to surface-induced disorder and segregation [21,24]. Both effects can be related to a change in the effective pair interactions between the different elements due to the decreased number of surface bonds which reduces the driving force of order or may induce other atomic arrangements which are not possible in the bulk. Consequently, it is natural to question, whether a completely ordered L1_0_ arrangement will be the stable ground state structure in the desired size regime [25–27]. Corroborating evidence comes from high resolution transmission electron microscopy (HRTEM) which shows that multiply twinned morphologies such as icosahedra and decahedra occur already at particle sizes around 6 nm in gas phase experiments [28–29]. These morphologies consist of several strained twins – twenty in the case of Mackay icosahedra and five in the case of decahedra. Although the twins may be perfectly L1_0_ ordered, they will not exhibit a significant uniaxial magnetocrystalline anisotropy because of the different crystallographic orientations of the individual twins in the particle.

The evolution of multiply twinned morphologies has been traced back to a competition between surface and volume energy contributions, which vary with particle size. This can be understood by means of a phenomenological third order polynomial law which expresses the binding energy as a function of the lateral system dimension, i. e., the third root of the system size *N* (for a discussion, see, e. g., [30]):

[2]
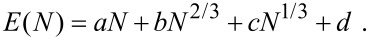


The coefficients *a*, *b*, *c*, and *d* describe the contributions to the binding energy arising from the particle volume, the facets, the edges and the vertices, respectively. They account for the shape of the particle, internal strains and interfaces and, of course, the materials bulk properties themselves. With decreasing system size, the coefficients *b*, *c* and *d* become one after the other important and it is straightforward to conceive that a morphology which can come up with a larger fraction of higher coordinated surfaces (and thus lower surface energy) due to twinning may become competitive with single-crystalline structures, which lack (energetically unfavorable) internal interfaces and strain in the volume part. This has been studied in depth for empirical models [30–34]. However, due to the complexity of the electronic interactions especially in magnetic materials, only parameter free first principles methods within the framework of density functional theory [35], which take into account materials properties on the electronic level, can be expected to provide useful theoretical predictions for a novel material combination with systematically improved properties. For real materials, such as the ones under consideration, the critical magnitude of the surface to volume ratio leading to crossover effects between different geometries can be expected to be in the range from a few hundred to several thousands of atoms. One suitable way to control the shape at a given size is by designing the ratio of the surface energy of different faces. Experimentally this may be achieved by tailoring the preparation conditions, e.g, by choosing suitable ligands in wet-chemical approaches [36–37]. Other authors suggest tackling the kinetics of the ordering processes and structure formation, e.g., by irradiation [38–41]. Both approaches are difficult to model on the basis of first principles calculations. Alternatively, one can try to increase the energy related to internal lattice defects, such as twin boundaries, by deliberate design of the alloy composition. This could effectively disfavor multiply twinned morphologies, while the resulting trends can be monitored on the electronic level in the framework of large scale density functional theory calculations. At this point, it should be kept in mind that segregated and multiply twinned morphologies may open up other fields of application. Core–shell structures are specially of interest, since enriching the catalytically active material (e. g., Pt or Pd) at the surface may reduce cost while the magnetic core provides another possibility for further manipulation [42]. In addition, the formation of an Pt-enriched shell may protect the Fe from oxidation [43].

A first step in the prediction of new materials for a specific purpose is to establish systematic trends between different alloys, which allow the energetic preference of a given morphology by a given material to be understood. By selecting components from suitable binary systems, systematic variation of the composition under addition of a ternary component can be attempted. The theoretical determination of ternary phase diagrams is an extremely demanding task for bulk systems and it becomes even harder if the size dependence must be accounted for as an additional variable. An important first step in this direction is thus to characterize changes in the energetic order of paradigmatic morphologies in binary alloys, which take place if one of the components is completely replaced by another element. A survey of such an effort covering 3d–5d alloys with elements in the vicinity of Fe–Pt in the periodic table has recently been undertaken by one of the authors [44]. The purpose of the current work is the extension to a ternary alloy in one specific case by means of large scale ab initio total energy calculations in the framework of density functional theory. For representative system sizes in the range of a few nanometers, where the surface-to-volume ratio is balanced and competitive effects should be expected such calculations are nowadays feasible on state-of-the-art supercomputer hardware such as the IBM Blue Gene/P at Forschungszentrum Jülich. The calculations presented here mainly concentrate on one size, 561 atoms, which corresponds to a diameter of about 2.5 nm. Clusters of this size possess a fraction of 45% surface atoms characterized by a reduced coordination in the first neighbor shell and are thus predetermined to monitor the competition between surface and bulk contributions with changing valence electron number.

## Computational

The calculations were carried out using the Vienna Ab-initio Simulation Package (VASP) [45], which expands the wavefunctions of the valence electrons into a plane wave basis set. The interaction with the nuclei and the core electrons is described within the projector augmented wave (PAW) approach [46] which yields an excellent compromise between speed and accuracy. For the accurate description of structural properties of ferrous alloys, the use of the generalized gradient approximation (GGA) for the representation of the exchange–correlation functional is mandatory. In the present work, the formulation of Perdew and Wang [47–48] in connection with the spin interpolation formula of Vosko, Wilk and Nusair [49] was used. Since the objects under consideration are zero-dimensional and thus non-periodic, the *k*-space sampling was restricted to the Γ-point in combination with Gaussian Fermi surface broadening. Its width was initially chosen as 50 meV and subsequently reduced to 10 meV. The description of the electronic properties with a plane wave basis requires a periodic setup. Thus all clusters were placed into a supercell, which requires a sufficient amount of vacuum separating the periodic images. The size of the cell was chosen such that a separation of of around 9 Å could be maintained. In order to restrict the numerical demands, a medium cutoff for the plane wave energy of *E*_cut_ = 270 V was used. For the same reason, only the electrons in the partially filled 3d and 4s shells were treated explicitly as valence electrons for the 3d elements, and the corresponding restriction was also made for the 4d and 5d elements. This has proven to be a reasonable compromise in a recent ab initio study of the lattice dynamics of ordered Fe rich alloys with Pt group elements [50]. For a few selected isomers, single-point calculations with an increased value *E*_cut_ = 335 eV were carried out for comparison. The energy differences between the isomers turned out to change by less than 0.1 meV/atom, which is far better than the overall accuracy in the order of several meV/atom that can usually be expected for calculations of this type.

A scalar relativistic formulation of the Hamiltonian was employed throughout. Thus within this work, only spin moments are reported omitting the orbital contributions, which might become sizeable in small particles and at the surfaces. The geometrical optimizations were carried out on the Born–Oppenheimer surface using the conjugate gradient method. The structural relaxations were stopped when the energy difference between two consecutive relaxations was less than 0.1 meV, leading to a convergence of forces down to the order of 10 meV/Å. The symmetrization of wavefunctions and forces was consistently switched off in all calculations.

The systematic search for the most stable structures of a given cluster size and composition involves the systematic scan of the potential energy surface, which is practically unfeasible from first principles for the system sizes under consideration. Therefore, the comparison is restricted to a pragmatic choice of selected morphologies, the so-called magic-number clusters. These have proven to be a good starting point as they appear to be particularly stable for the late 3d elements [51], especially Ni and Co. Their size *N* is can be expressed by the number *n* of closed geometric shells:

[3]
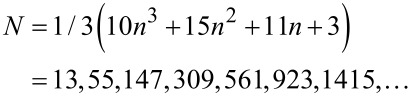


Magic-size clusters allow a comparison of several paradigmatic geometries to be made: Cuboctahedra with a face centered cubic (fcc) or tetragonal structure, Mackay icosahedra [52] and Ino decahedra [53]. In the present study, only icosahedra and cuboctahedra are considered. The latter only with perfect L1_0_ order (but with different magnetic configurations), while for the icosahedra both ordered and disordered arrangements are taken into account. The composition of a perfectly ordered binary cluster motif generally varies with system size, atomic arrangement and termination of the surfaces. In our studies it was kept fixed to allow for the construction of single crystalline structures with a perfect L1_0_ arrangement of 3d and 5d layers, and Pt covered [001] surfaces. In the present case, a cluster of *N* = 561 atoms contains 265 3d and 296 5d atoms. In order to describe ternary compositions, Fe atoms were replaced by Mn on randomly chosen 3d sites. This is a reasonable starting point for the investigation of ternary 3d–5d alloys, since both limiting binary cases exhibit L1_0_ order and the ordering tendencies in terms of 3d and 5d elements can be considered to be much stronger due to the large difference in size and electronic properties than for the 3d elements Fe and Mn, which are neighbors in the periodic table. Again, the configurations were kept fixed for all calculations with the same morphology type and composition. This leaves aside possible effects due to segregation of one elemental species to the surface or internal interfaces as twin boundaries.

## Results and Discussion

The size dependent evolution of morphologies of near stoichiometric Fe–Pt, Co–Pt and also partially Mn–Pt particles has been subject of recent publications by the authors [26–27,54–56]. A brief summary of the main results will therefore be given below.

In the case of magic number Fe–Pt clusters with up to seven close geometric shells, the most favorable morphology found so far has been identified as an icosahedron with onion-ring-like alternating Fe and Pt shells and Pt covered (111) facets. The arrangement of the atomic species within the cluster can also be understood as an individual L1_1_ ordering of the twins. The bulk L1_1_ order is characterized by an alternation of close packed 3d and 5d layers along the space diagonal (in contrast to the layering along the *c* axis in L1_0_). This is found in bulk only for CuPt [57].

The onion-ring structure is, for diameters around 2.5 nm (*N* = 561), lower by 30 meV/atom than the L1_0_ ordered single crystalline structure, which we would rather prefer for data storage applications due to its allegedly large magnetocrystalline anisotropy energy. The stability of the multiply twinned structures is even greater in CoPt, the second candidate discussed in the introduction. Here, segregated core–shell structures are the dominating lowest energy morphologies for *N* = 561 being up to 120 meV/atom lower than the L1_0_ ordered isomers. Also the aforementioned onion-ring structure turns out to be much more favorable than in the Fe–Pt case. This trend can be understood by considering bulk and surface contribution to Equation 2. Ab initio calculations predict a nearly linear increase of the energy difference between L1_0_ and L1_1_ structure of equiatomic alloys between Pt and 3d transition metals with decreasing valence electron concentration *e*/*a* [58]. While the L1_1_ phase is energetically lowest in CuPt, the L1_0_ phase is clearly favored for CoPt and even more so for FePt and MnPt. On the other hand, recent surface energy calculations [55] have shown that L1_1_ FePt and CoPt alloys possess extremely low surface energies for purely Pt covered (111) surfaces. The corresponding values are significantly lower than the contribution for all other low index surfaces that have been obtained for the L1_0_ arrangement. Modeling the competition of the surface and bulk energy contributions by varying with cluster size, in keeping with Equation 2, yields appropriate trends in the cross-over sizes [55] which are furthermore in good agreement with the ab initio cluster calculations. We thus conclude that for FePt and CoPt the energy gain from the surface contribution is large enough to stabilize the L1_1_ order in the particle core at sufficiently small particle sizes and also compensates the energy which is required for the formation of twin boundaries. In larger particles, however, some kind of hybrid arrangement should be expected, which will allow for L1_0_ order in the particle core and a change to an onion-ring arrangement in the surface layers. Onion-ring and hybrid morphologies have been considered for other alloys [59–61] as well as for Fe–Pt in the context of surface induced disorder [24,62–64]. This has been verified very recently in a combined ab initio and Monte Carlo approach [65], while representations of hybrid arrangements have also been found to be competitive with the layer-wise and shell-wise ordered morphologies in a recent large scale first principles approach [56].

It is known from several first principles investigations [66–69] that FePt in its L1_0_ phase is at the brink of magnetic instability and exhibits a latent tendency to form a layer-wise antiferromagnetic (AF) spin order. This is accompanied by a slight increase of the tetragonal distortion with respect to the fcc lattice constant. Such a phase has not been observed in experiment so far, but may become apparent if potentially antiferromagnetic components such as Mn are added. According to the suggestion of Brown and coworkers [67], the suppression of the magnetic instability in pure FePt can be ascribed to the incomplete order of the experimental samples which introduces Fe atoms into the Pt layers. This modifies the effective interlayer coupling and mediates an indirect ferromagnetic (FM) interaction between the adjacent 3d layers, which overrides the smaller direct antiferromagnetic coupling across the Pt layer. The validity of this model has been verified in large scale first principles calculation of a partially disordered L1_0_ cluster [68]. Furthermore, it has been argued that spin–orbit interaction provides further stabilization of the FM phase [66,70].

The element resolved electronic structure provides another way to obtain a qualitative understanding of the chemical trends on the energetic order of morphologies [26–27,54]. A comparison of the densities of states of the Fe–Pt, Co–Pt and Mn–Pt L1_0_ cuboctahedra and onion-ring icosahedra, which was carried out by the authors in [27], reveals that for both morphologies the change of the electron number induces a nearly perfectly rigid shift of the 3d minority spin states, while the 5d and the majority states remain nearly unaffected. This makes the distribution of the minority spin 3d states in the vicinity of the Fermi level a decisive factor for the evolution of the stability of the structures with composition. When replacing Fe by Co, the additional d electrons of Co necessarily fill up the minority channel, because the majority spin states are occupied. This shifts the contributions of Co to lower energies. The corresponding shift, however, is larger for the onion-ring icosahedron, since the density of the L1_0_ minority 3d states encounter a steep increase above *E*_F_ (which is less pronounced for the multiply twinned structures) while the electron densities at the Fermi level *E*_F_ are nearly the same for the isomers. This results in a different contribution to the band energy with respect to the L1_0_ reference. Following this simple picture, the opposite might be expected to happen in Mn–Pt, as 3d electrons here are removed.

**Figure 1:**
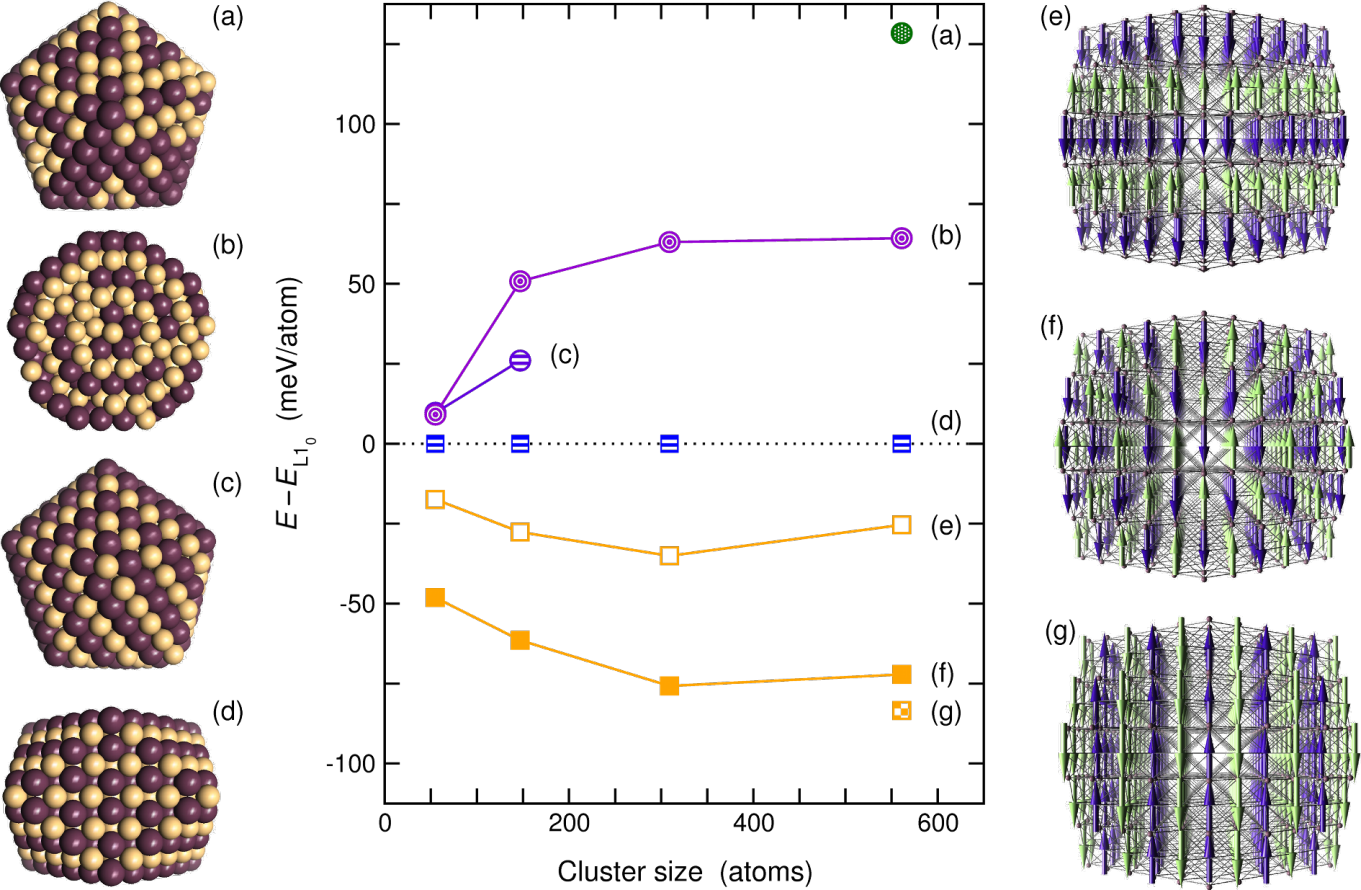
Energies of Mn–Pt clusters of various morphologies and sizes. The energy reference is marked by the L1_0_ cuboctahedron. The letters in parentheses refer to the corresponding structures which are depicted at both sides of the diagram (only the structures with *N* = 561 atoms are shown). These are: (a) the disordered icosahedron, (b) the onion-ring icosahedron in cross section, (c) an ordered icosahedron, (d) the ferromagnetic L1_0_ cuboctahedron, (e) an antiferromagnetic cuboctahedron with layered spin arrangement perpendicular to the *c* axis, (f) a staggered antiferromagnetic cuboctahedron and (g) an antiferromagnetic cuboctahedron with spin arrangement layered parallel to the *c* axis. Orange spheres (or, respectively, blue and green arrows) refer to Mn atoms, purple spheres to Pt. Colors and symbols: Shaded green circles denote disordered icosahedra (a). Triply nested circles refer to icosahedra with shell-wise ordering tendencies (b), hatched blue to violet symbols the ordered structures (c) and (d). Orange symbols denote L1_0_ cuboctahedra with antiferromagnetic ordering of the 3d moments (e)–(g). The lines are only guides for the eye.

This seems indeed to be the case, if Fe is completely replaced by Mn. Figure 1 demonstrates that the alternating icosahedron is located approximately 65 meV/atom above the 561-atom L1_0_ cuboctahedron (as compared to ≈ 30 meV/atom below for FePt). The ferromagnetic, ordered icosahedron, which is nearly degenerate for Fe_265_Pt_296_, has become unstable in the Mn–Pt system for sizes above 147 atoms. During the geometric optimization procedure it transforms downhill to a perfect L1_0_ cuboctahedron. This proves that the Mackay path is a also realistic transformation path for binary magic-number transition metal systems but, in addition, assures that a simple energy minimization effectively helps to discriminate the most important classes of structures. It should be noted at this point that the simple rigid band picture does not hold quite as nicely here as for the replacement of Fe by Co. As shown in [27], for the onion-ring structure a completely ferromagnetic configuration could not be obtained leading to antiferromagnetic alignment of parts of the Mn spins with respective contributions in the majority spin channel above the Fermi level and in the minority channel below, which alter the overall shape of the total DOS.

This points out the major drawback in using Mn as stabilizing agent for L1_0_ particles for magnetic recording purposes: Its preference for antiferromagnetic ordering, which is well known for the bulk system and also present in nanoparticles. The lowest energy isomer shown in Figure 1 is an L1_0_ cuboctahedron with staggered antiferromagnetic arrangement of the spins within the Mn layers. Thus, the restriction to binary systems by systematic exchange of one element does not yield substantial improvements with respect to their applicability for data recording purposes. In fact, the only component identified so far in our studies that raises hope to suppress substantially multiply twinned structures by co-alloying is Mn. However, as Mn might elicit the latent antiferromagnetic tendencies which are present in pure Fe–Pt as discussed above, an unfavorable change of the magnetic structure with increasing Mn content might be the consequence. On the other hand, it has been shown from fully relativistic electronic structure calculations of the bulk alloy that in the ferromagnetic phase small admixture of Mn will increase the magnetocrystalline anisotropy energy [71], while Lai and Ho found for chemically prepared particles with diameters around 4 nm that adding Mn is beneficial for the coercivity, which the authors ascribe in the first instance to an improved L1_0_ order of their particles [72].

It is therefore of increased interest to take a look at this system in more detail. In the present study this was realized by exchanging a given fraction of Fe sites randomly by Mn, leaving the Pt sites untouched: The configurations of the Fe–Pt system served as a pre-optimized template. Afterwards, the clusters underwent an optimization of the ionic positions as in the other cases. Comparison is restricted here to L1_0_ cuboctahedra and onion-ring icosahedra with 561 atoms (265 3d metal and 296 Pt atoms). For the ordered L1_0_ clusters, different magnetic configurations were taken into account: The perfect ferromagnet, the staggered and layered antiferromagnet as well as a ferromagnetic configuration where the Mn spins are reversed with respect to the Fe spins. The icosahedra were always initialized with a ferromagnetic configuration, but again at the Mn-rich side several spins could not be prevented from flipping spontaneously.

The phase diagram of ternary Fe_1–_*_x_*Mn*_x_*Pt was examined experimentally in detail by Menshikov et al. [73] by means of X-ray and neutron diffraction measurements on a powder sample. The authors found that the alloy assumes a nearly, but not perfectly L1_0_ ordered tetragonal structure for all compositions. The degree of tetragonality strongly increases at low Mn content up to an equiatomic mixture of both elements and reaches finally values of *c*/*a* ≈ 0.92 for nearly pure MnPt. The authors describe the magnetic structure to evolve from a ferromagnet with an easy axis perpendicular to the Fe and Pt planes at *x* = 0 to a staggered antiferromagnetic structure with easy plane anisotropy in the range 0.25 ≤ *x* ≤ 0.5. On the Mn rich side, the orientation of the moments switches back to perpendicular to the antiferromagnetically ordering 3d planes. In between, the authors report for the low temperature range ferro- and antiferromagnetic regions with canted moments. Later, Meyer and Thiele [74] investigated the same system as epitaxial films grown on MgO. Their XRD (X-ray diffraction) data essentially confirmed the structural properties reported in [73] despite possible mechanical strains due to the thin film setup. Using a vibrating sample magnetometer for saturation magnetization and hysteresis loop and X-ray magnetic circular dichroism (XMCD) to obtain the element resolved orientation of the moments, the authors observed a linear decrease of the average magnetization with increasing Mn-content, which finally vanishes completely around *x* = 0.5. From their XMCD data, the authors conclude that Mn and Fe predominately align in an antiparallel fashion over the whole composition range and thus rule out a composition-dependent sign change in the Fe–Mn magnetic exchange constant which was postulated by Menshikov [73,75].

**Figure 2:**
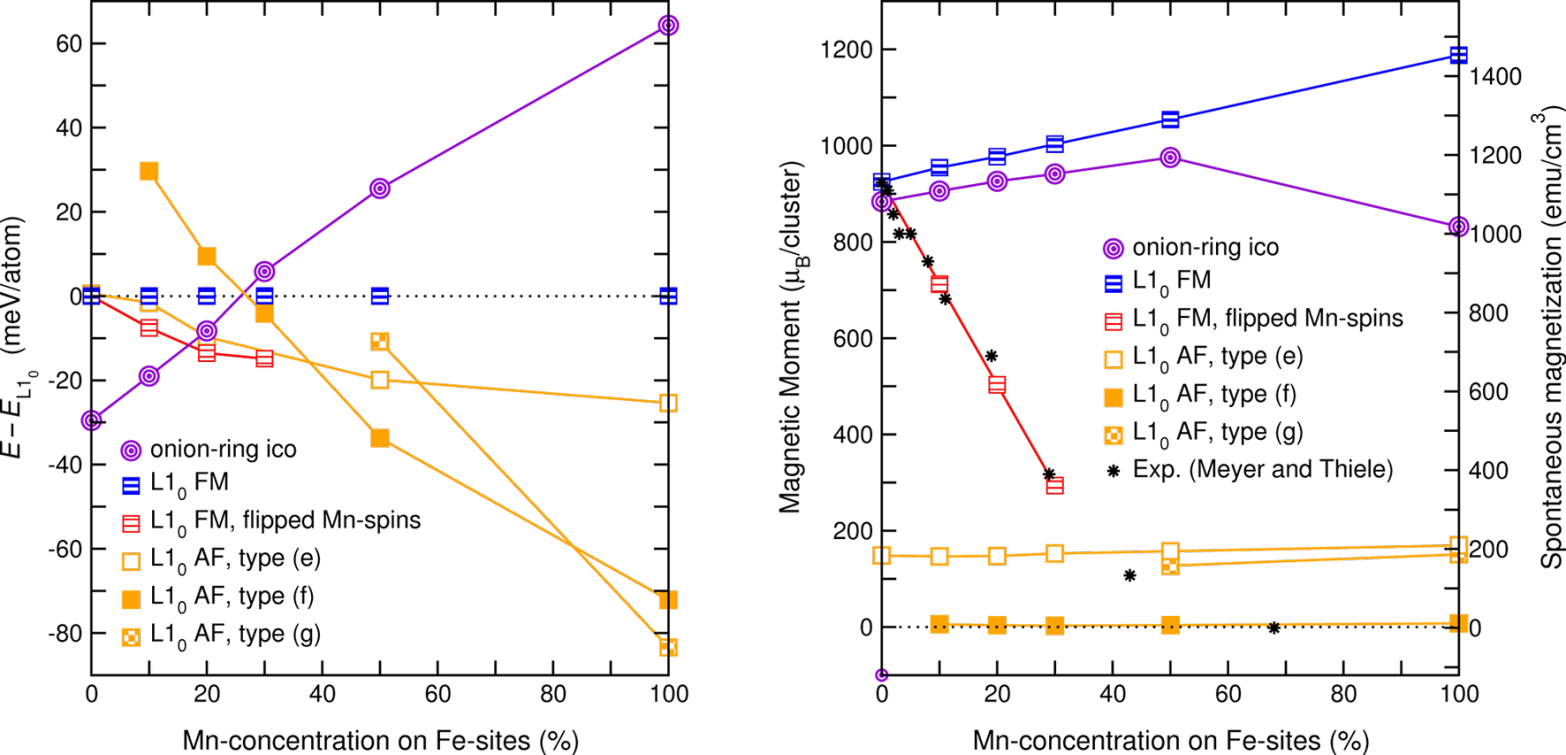
Energetic order (left panel) and magnetization (right panel, left scale) of ternary 561 atom Fe–Mn–Pt clusters with different morphologies and magnetic structures as a function of composition (Fe sites randomly replaced by Mn). Blue squares denote the ferromagnetic (FM) and perfectly ordered L1_0_ isomer (d), which again is chosen as reference for each composition, violet nested circles the alternating icosahedron (b). Orange square refer to antiferromagnetic (AF) clusters: Filled symbols to a staggered antiferromagnetic configuration (f), open symbols to a layerwise alternating arrangement of ferromagnetic 3d layers parallel to the *c* axis (e), half-filled symbols to an antiferromagnetic arrangement layered perpendicular to *c* (g). Red symbols denote a ferromagnetic configuration (d), where Fe and Mn moments point in opposite directions. The black stars refer to the experimental saturation magnetization (right scale) obtained by Meyer and Thiele [74].

The left side of Figure 2 depicts the energetic order of the clusters as a function of the Mn concentration. Random replacement of up to 20% of the Fe sites by Mn decreases the stability of the multiply twinned structure significantly, such that a crossover with the ferromagnetic L1_0_ cuboctahedron already occurs around 25 atom % Mn. On the other hand, the possibility of different antiferromagnetic structures at either end of the composition range as well as the possible presence of competing ferro- and antiferromagnetic exchange interactions must be taken into account in the ternary system. Therefore, also the layered and staggered antiferromagnetic configurations were included in the comparison. In addition, in the Fe-rich part, a ferrimagnetic setup was considered with Mn spins entirely aligned antiparallel to the ferromagnetically ordered Fe-spins. In fact, up to *x* 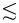 30, this configuration represents the most favorable of the cuboctahedral isomers and has the lowest energy of all structures under consideration for *x* 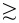 17.

Because of the latent antiferromagnetic tendencies in FePt, a small fraction of Mn atoms will make the layer-wise antiferromagnetic cuboctahedron more favorable than the ferromagnetic reference, which consequently turns out very close in energy to the ferrimagnetic isomer. Since in the layered antiferromagnet, the Mn atoms were initially parallel to the Fe atoms, one might expect an analogous lowering of the energy, which could make this configuration the most favorable L1_0_-type structure in this concentration range. However, as discussed above, for binary Fe–Pt such a configuration will effectively be suppressed by a small amount of disorder in the system, due to Fe or Mn occupying a Pt site and mediating an effective ferromagnetic interlayer coupling. On the other hand the staggered antiferromagnet, which is the most favorable isomer for compositions with more than 40% of Mn atoms, is not affected by this kind of disorder. The energetic order of the L1_0_ coincides very well with the experimental saturation magnetization obtained by Meyer and Thiele [74], which is shown by the black stars in the right panel of Figure 2. It obeys essentially the same concentration dependence as the ferrimagnetic isomer for *x* 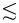 40 and vanishes when the staggered antiferromagnet becomes the ground state. The layered spin configuration exhibits, in spite of its AF nature, a finite spin moment throughout. This results from the uneven number of 3d layers in the 561-atom cluster with five complete geometric shells. The consequence is that due to symmetry reasons the moments within the outermost 3d layers are aligned in the same direction and therefore do not compensate each other. The staggered AF on the other hand has a nearly vanishing total spin moment apart from a residual value of a few Bohr magnetons, which results from a small number of uncompensated spins in the edge and corner parts. Indeed, low temperature ferromagnetism in combination with exchange bias effects originating from uncompensated surface spins has been observed experimentally in annealed binary MnPt nanoparticles with diameters between 2.3 nm and 4.1 nm [76]. The kink of the otherwise linear evolution of the spin magnetic moment of the icosahedral cluster at large Mn content is due to the barely stable FM configuration in Mn rich particles, which is reflected in spontaneous spin-flips decreasing the magnetization.

A very interesting aspect of this system is that there is a crossover between various magnetic and structural phases between 15 and 50% Mn on Fe sites. While the crossover point between icosahedra and cuboctahedra is determined by the competition between surface and volume contributions to the total energy and thus strongly size dependent, this is far less the case for the L1_0_ isomers with different magnetic structures, as here the surfaces are of identical composition and thus play a much less dominant role.

**Figure 3:**
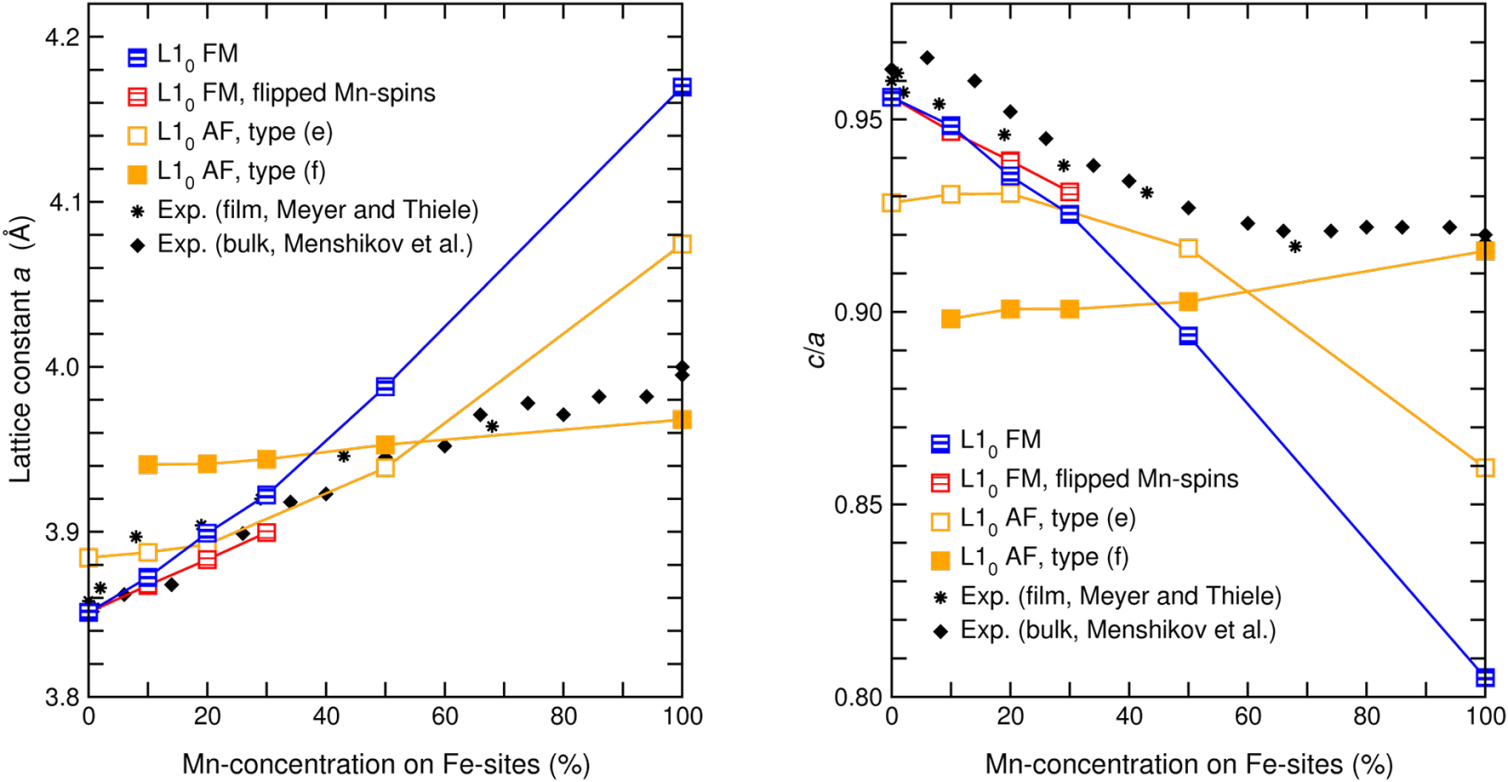
Lattice constant (left panel) and *c*/*a* ratio (right panel) of ternary 561-atom Fe–Mn–Pt clusters with different morphologies and magnetic structures as a function of the composition. Symbols as in Figure 2. The lattice parameters were obtained by averaging the interlayer distances in the respective directions. The values are compared with the experimental XRD data obtained at room temperature by Meyer and Thiele [74] for thin films (black stars) and Menshikov et al. [73] for bulk powder samples (black diamonds).

In order to allow a direct comparison of structural cluster properties with bulk and thin film experiment, the distances between the layers and interlayer distances have been calculated from the averaged projections of the position vectors in the direction of the face normal. These yield the corresponding interlayer distances, which are finally averaged to obtain the effective lattice parameters *a* and *c* of the L1_0_ type clusters. A comparison of the lattice constant *a* between calculation and the experimental values of Meyer and Thiele [74] and Menshikov et al. [73] is provided in Figure 3 (left panel). In contrast to the other magnetic isomers, which are characterized by a considerable change of *a* with *x*, the lattice constant of the staggered AF remains nearly constant over the whole concentration range. This trend agrees well with the experimental observation in the Mn rich part (*x* 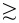 50), while for *x* 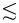 30 the measured values coincide nicely with the steeper slope of the ferro- and ferrimagnetic isomers, which indicates at least one change of the magnetic structure in between. A similar picture is obtained for the composition dependence of the tetragonality, as given by the ratio *c*/*a* (Figure 3, right panel). Here again, the *c*/*a* ratio of the staggered AF undergoes only a slight variation, while for the FM isomer a strong decrease is observed finally reaching a value as low as *c*/*a* = 0.81 for MnPt. At the Fe rich end this is also the case for the ferromagnetic configuration with inverted Mn spins, which, however, shows with increasing Mn content a less strong variation compared to the pure FM case.

## Conclusion

The results demonstrate, that the addition of Mn to the Fe–Pt system by trend stabilizes antiferromagnetic order. Nevertheless, at small Mn concentrations, magnetically inhomogeneous states with antiparallel Mn moments are competitive which are still ferromagnetic at large. Although the antiferromagnetic admixture to a ferromagnetic L1_0_ configuration must be expected to decrease the performance of Fe_1–_*_x_*Mn*_x_*Pt nanoclusters in data recording applications, such a configuration might provide a suitable compromise as it improves the structural properties. To answer this question finally, further investigations are necessary taking into account the impact of increased magnetic disorder on finite temperature properties and spin–orbit interaction in the framework of fully relativistic first principles calculations. It might also be necessary to investigate how far increased segregation of one species to surface and interfaces could affect the energetic order of the paradigmatic morphologies.

Finding various phases with different structural and magnetic properties in a close interval of composition and energy gives rise to the hope that this material may allow the selection of specific magnetic or structural modifications with a fairly small energetic effort, which could be provided by an external magnetic field. In this respect, it looks promising that the latent tendencies of FePt for a layered AF structure is in fact stabilized by the addition of a few percent of Mn. FM and AF configurations show a considerable difference in their *c*/*a* ratio while the energy differences are small. However, one must keep in mind that an effective device would require extremely high degrees of order of the active material, which might be particularly difficult to realize on the nanoscale, as outlined in the introduction. However, interesting crossover effects can also be expected in the region 0.3 ≤ *x* ≤ 0.6, where the experimental *c*/*a* and the lattice parameter *a* change their slope and different magnetic structures become competitive in energy. If composition and degree of order are carefully tuned, it might be possible to select the ferro or ferrimagnetic phase by an external magnetic field, while the ground state is still AF. In fact, Menshikov et al. [73] demonstrated in their experiments, that an external magnetic field can induce a magnetization at finite temperatures in the vicinity of the Néel temperature, which decays again towards high as well as towards low temperatures. The authors explain this fact with the presence of FM clusters with possible diameter of 5–10 nm in an otherwise AF matrix. From the present results a spin glass like ground state must also be considered. This question might be resolved in a later stage by additional simulations with statistical models making use of ab initio exchange parameters, which can be easily determined in bulk calculations. This, however, is beyond the scope of the present work. Nevertheless, the fact that a magnetic field can induce a magnetized state, which, however, does not necessarily relate to a higher degree of magnetic order, i. e., a lower magnetic entropy, raises the hope that a suitably designed material might exhibit a significant inverse magnetocaloric effect and thus be of potential interest for magnetic cooling purposes. In combination with the corresponding changes in lattice parameters and atomic volumes (the latter is substantially larger for the FM case), which can be inferred from Figure 3, the entropy change associated with the magnetic transition might become sizeable. As the changes in lattice parameter and *c*/*a* can be in the order of a few percent, it might also be worthwhile to explore in more detail, whether corresponding magnetic field induced structural changes can be used for magnetomechanical devices on the nanoscale, which could, e.g., consist of Fe_1–_*_x_*Mn*_x_*Pt nanoparticles embedded in an organic matrix.
